# Idiopathic pulmonary fibrosis from a multiscale mechanobiology perspective: Mechanisms and future therapeutic prospects

**DOI:** 10.1016/j.isci.2026.115335

**Published:** 2026-03-12

**Authors:** Tingting Xia, Xi Wang, Zhao Yang, Runze Zhao

**Affiliations:** 1 Department of Cell Biology, School of Basic Medical Sciences, Suzhou Medical College of Soochow University, Suzhou 215101, China; 2 Medical 3D Printing Center, Orthopedic Institute, Department of Orthopedic Surgery, The First Affiliated Hospital, School of Basic Medical Sciences, Interdisciplinary Innovation Center for Nanomedicine, MOE Key Laboratory of Geriatric Diseases and Immunology, Suzhou Medical College, Soochow University, Suzhou, Jiangsu 215000, China; 3 Department of Respiratory Medicine, Suzhou Hospital, Affiliated Hospital of Medical School, Nanjing University, Suzhou, Jiangsu 215153, China; 4 Center of Translational Medicine, The Fourth Affiliated Hospital of Soochow University, Medical Center of Soochow University, Suzhou Dushu Lake Hospital, Suzhou, Jiangsu 215123, China

**Keywords:** mechanobiology, disease

## Abstract

Idiopathic pulmonary fibrosis (IPF) is a fatal interstitial lung disease that induces irreversible fibrosis and architectural remodeling. Traditional inflammation-based theories fall short in explaining its pathological processes, regional heterogeneity, and spatially biased lesion distribution. Recent studies have highlighted the critical role of biomechanical microenvironment, ranging from the molecular and cellular level to the whole-organ scale, in driving fibrotic progression. This review adopts a multiscale biomechanical perspective for understanding IPF pathogenesis, integrating molecular, cellular, tissue, and organ-level mechanisms. We summarize recent advances in IPF research from five key biomechanical perspectives: mechanotransduction, mechanical memory, extracellular matrix (ECM) stiffening, strain-induced fibroblast activation, and the spatial propagation of fibrosis. We further explore therapeutic strategies targeting mechanical signaling pathways and discuss the integration of machine learning and physics-informed neural networks (PINNs) for interpretable, physiology-constrained modeling. This review aims to provide a new mechanobiological perspective for understanding and intervening in IPF.

## Graphical abstract

**Figure:**
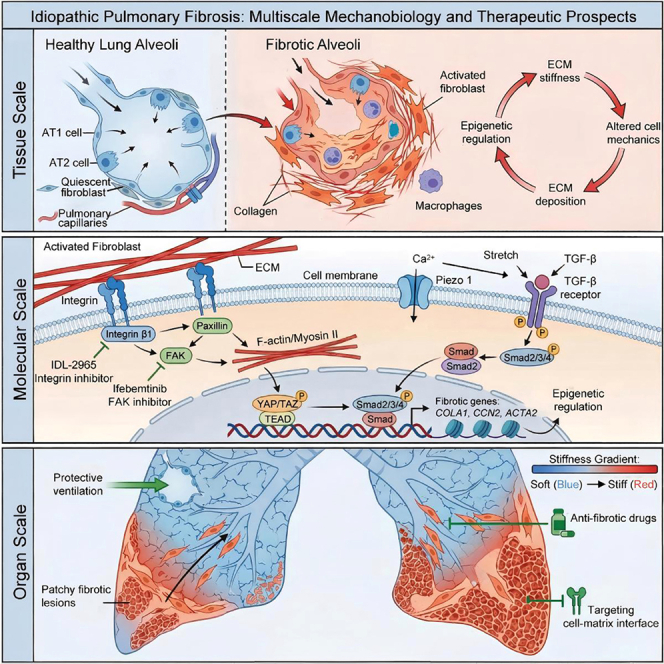

## Introduction

Idiopathic pulmonary fibrosis (IPF) is a progressive interstitial lung disease characterized by persistent alveolar collapse, excessive interstitial collagen deposition, and reduced lung compliance, ultimately resulting in severe impairment of gas exchange. According to the American Thoracic Society (ATS) 2023 clinical guidelines, the median survival of patients with IPF is typically only 2–3 years, shorter than some advanced solid tumors.^1^^,^^2^^,^^3^^,^^4^^,^^5^ Currently approved antifibrotic drugs, such as pirfenidone, nintedanib, and nerandomilast, can only alleviate the decline of lung function but rarely achieve true structural reversal.^6^^,^^7^ This calls for better mechanistic understanding and more effective therapeutic targets beyond current models.

Over THE past decades, IPF research has largely focused on inflammatory responses and profibrotic factors, especially transforming growth factor β (TGF-β) driven signaling pathways.^8^ Well-studied axes include TGF-β/SMAD, MAPK, and PI3K/AKT, elucidating molecular bases for fibroblast activation and abnormal extracellular matrix (ECM) deposition.^9^^,^^10^^,^^11^ However, the classic “inflammation-cytokine-matrix deposition” model struggles to explain persistent clinical and imaging puzzles: Why does IPF predominantly originate in the subpleural basal lung regions with a characteristic spatial distribution? Why do fibrotic lesions display honeycomb-like structures instead of uniform matrix deposition? Why do many patients exhibit significant structural damage even in the absence of overt inflammation^12^^,^^13^?

With rapid advances in mechanobiology, high-resolution imaging, and multiscale computational modeling, researchers increasingly recognize that complex mechanical microenvironment changes within lung tissue may be the “hidden driver” initiating and advancing IPF.^14^^,^^15^ The lung expands and contracts continuously with each breath. During this cyclic motion, its structural components, including alveoli and blood vessels, are subjected to various mechanical forces, such as stretch, shear, and compression. These stresses maintain tissue homeostasis under normal physiology; their disruption may represent the critical initiator triggering fibrosis.^15^

**Figure 1:**
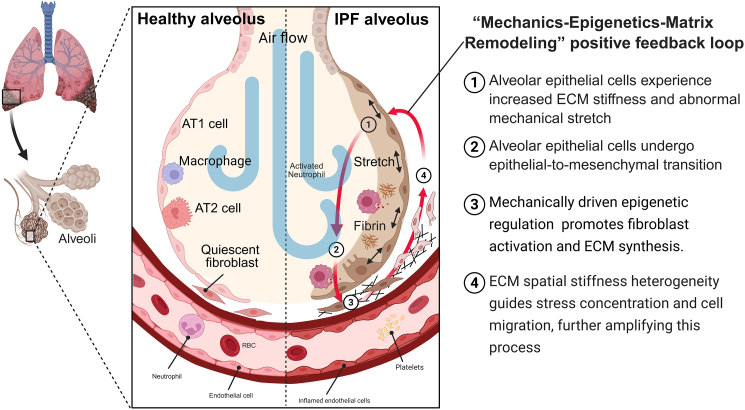
The pathogenesis of the mechanobiological hypothesis of IPF: a “mechanics-epigenetics-matrix remodeling” positive feedback loop Compared with healthy alveoli, alveolar epithelial cells in IPF are exposed to a stiffer ECM and subjected to abnormal mechanical stretch. Under this pathological mechanical microenvironment, these epithelial cells are transformed to mesenchymal phenotypes (EpMT), which further increases matrix stiffness. In addition, resident macrophages can transform into an inflammatory phenotype, thereby secreting inflammatory factors to exacerbate fibrosis. The increased stiffness of the substrate can persistently stimulate fibroblasts to an activated state through epigenetic regulation, leading to continuous ECM secretion and a global stiffening of the lesion areas. These lesion areas-induced spatial stiffness heterogeneity leads to local stress concentration and directs fibroblast durotactic migration toward stiffer areas. Ultimately, this mechanically reinforced microenvironment exacerbates alveolar epithelial dysfunction, forming a self-amplifying positive feedback loop. This figure was created with BioRender software.

From this emerging perspective, IPF should not only be seen singly as a cytokine-driven inflammatory disease but also as a progressive disorder driven by mechanical perturbations, structural feedback amplification, and eventual tissue destabilization. Specifically, alveolar epithelial cells (AECs) exposed to stiff ECM or abnormal stretch undergo epithelial-to-mesenchymal transition (EpMT), which promotes matrix synthesis.^16^^,^^17^ Fibroblasts develop a “mechanical memory” on stiff substrates, maintaining activation even when returned to softer environments.^18^^,^^19^ Spatial heterogeneity of ECM stiffness guides stress concentration and cell migration, facilitating the expansion of fibrotic foci. Additionally, regional strain generated by respiratory motion further amplifies these processes, leading to the preferential involvement of the lung bases and accelerated disease progression.^20^^,^^21^^,^^22^ Collectively, these phenomena form a typical “mechanics-epigenetics-matrix remodeling” positive feedback loop, exhibiting strong multiscale coupling from molecular to cellular, tissue, and organ levels (Figure 1).

Based on current advances, this review aims to systematically delineate the key mechanobiological mechanisms underlying IPF pathogenesis from a multiscale perspective. We discuss four hierarchical levels: microscopic-how AECs and fibroblasts sense and respond to mechanical stimuli; mesoscopic-how ECM stiffness heterogeneity drives spatial fibrotic expansion; macroscopic-how respiratory mechanics and regional lung strain influence pathological distribution; and translational-mechanical targeted therapies and clinical prospects of multiscale computational models.

By integrating mechanobiological regulatory mechanisms in IPF initiation and progression, we seek to bridge gaps between molecular insights and imaging phenotypes, providing novel directions for precision diagnosis and individualized intervention.

## Molecular scale: the initiating role of mechanosensitive molecules

### Macrophage mechanotransduction in early fibrotic responses

In the early stage of IPF, the responders to the mechanical microenvironment changes are individual cells within the alveolar network, including AECs, lung fibroblasts, and resident macrophages. These cells continuously sense different mechanical cues, such as matrix stiffness, stretch, and strain, through special mechanoreceptors. Through mechanotransduction pathways, these cells perform essential functions such as gas exchange, matrix maintenance, and immune surveillance. Among them, macrophages play a pivotal role in facilitating the early IPF progression through translating aberrant mechanical signals into biochemical signals. Mechanical properties of the ECM, including stiffness, topography, and interstitial flow, directly influence macrophage polarization through mechanosensitive pathways such as Yes-associated protein (YAP)/TAZ, integrin-focal adhesion kinase (FAK), and NF-κB signaling.^16^^,^^17^^,^^18^^,^^19^^,^^20^^,^^21^^,^^22^^,^^23^^,^^24^^,^^25^^,^^26^ Although several studies report that increased matrix stiffness promotes pro-inflammatory M1 polarization, others demonstrate stiffness-induced M2 polarization, highlighting the complexity of mechanotransduction and the downstream regulation of macrophage.^22^^,^^24^^,^^25^^,^^26^ Besides stiffness, interstitial fluid flow and spatial confinement further regulate macrophage polarization by modulating cytoskeletal organization, transcriptional programs, and epigenetic states via integrin/Src-STAT3/6 and actin MRTF-A dependent mechanisms.^27^^,^^28^ In addition, mechanosensitive ion channels, including Piezo and TRP family channels, can translate mechanical stimuli into calcium-dependent signaling that precisely regulates macrophage polarization.^26^^,^^29^^,^^30^^,^^31^^,^^32^^,^^33^

### Mechanosensing and signal transduction in alveolar epithelial cells

Alveolar type II epithelial cells (AT2) are pivotal for maintaining surfactant synthesis and alveolar integrity.^34^ AT2 cells act as resident progenitors of the alveolar epithelium, and their differentiation into alveolar type I cells (AT1 cells) represents a critical step in lung development and injury repair.^35^^,^^36^ Located on the alveolar walls, AT2 cells are the earliest to be directly exposed to cyclic stretch stresses caused by respiratory movements.^37^ Recent studies have revealed that this process is not driven solely by biochemical cues but is profoundly regulated by mechanical signals within the alveolar microenvironment.^38^ As a persistent physical stimulus during breathing, mechanical forces are transduced through a complex mechanotransduction machinery to directly instruct AT2 cell fate decisions, including proliferation, surfactant secretion, and differentiation toward the AT1 lineage.^39^

AT2 cells sense the mechanical cues through multiple molecular mechanosensors. First of all is the integrin-focal adhesion complex, which acts as a transmembrane bridge connecting the ECM and the intracellular actin cytoskeleton. This complex is recognized as the primary mechanosensor for sensing the mechanical properties of the ECM and transmitting tensile forces.^38^^,^^40^ During the differentiation of AT2 into AT1 cells, the integrin subtype expression shifts from the AT2-associated integrin αvβ6 to the AT1 characteristic integrin αvβ5, which is accompanied by the phosphorylation and activation of FAK.^38^ A study has demonstrated that this transition is indispensable for AT2-to-AT1 differentiation, as the inhibition of FAK activity markedly suppresses the capacity of AT2 cells to undergo AT2-to-AT1 differentiation.^38^ In addition, integrin β1-mediated cell-ECM interactions have been demonstrated to be a foundation of establishing a normal AT2-to-AT1 differentiation program; disrupting these interactions leads to defective alveolar repair.^41^

In addition, mechanosensitive ion channels, such as Piezo1/2 and TRPV4, can be activated directly when the mechanical tension is applied to the plasma membrane. These channels open rapidly and mediate rapid calcium (Ca^2+^) influx, thereby initiating downstream signaling cascades.^42^^,^^43^ This Ca^2+^ influx subsequently stimulates calcium/calmodulin-dependent kinases and related pathways, ultimately regulating cytoskeletal dynamics and the nuclear translocation behavior of YAP/TAZ.^44^ Accumulating evidence reveals that Ca^2+^ influx is essential for the stretch-induced differentiation of AT2 cells into the AT1 lineage.^42^ Notably, distinct channels appear to respond to different magnitudes of mechanical stimulation. For instance, TRPV4 is primarily involved in AT2-to-AT1 differentiation under physiological stretch, whereas Piezo2 responds to higher-intensity stretch corresponding to potentially injurious forces, and its sustained activation could lead to pathological consequences.^43^

Finally, the cytoskeleton and nuclear mechanosensors constitute the last responders of mechanotransduction. Besides being a passive consequence of mechanical stimuli, remodeling of the actin cytoskeletal network is an integral component of mechanical force-mediated signal transduction, transmitting the mechanical cues through the cytoskeleton to the nucleus. At the nuclear level, these mechanical signals regulate the activity of the transcriptional co-activators YAP/TAZ and modulate their interactions with the nuclear lamina and chromatin, thereby enabling precise control of the downstream gene expression.^45^^,^^46^

YAP and TAZ are key transcriptional co-activators downstream of the Hippo pathway, but their activity is strongly regulated by mechanical signals.^45^^,^^47^ Under the mechanical conditions of a stiff ECM or cyclic stretch, YAP/TAZ can translocate into the nucleus, binding to the transcription factors such as TEAD, which subsequently initiate a program of gene expression that promotes cell proliferation and differentiation, serving as a central switch driving AT2-to-AT1 differentiation.^48^^,^^49^ In contrast, when cells are cultured on a soft matrix or in the absence of mechanical stimulation, YAP/TAZ are retained in the cytoplasm and degraded via the ubiquitin-proteasome pathway, allowing AT2 cells to maintain their progenitor characteristics.^50^ A study further demonstrates that the specific deletion of YAP/TAZ in AT1 cells leads to their spontaneous reprogramming into AT2 cells, confirming their indispensable role in maintaining AT1 cell phenotype.^46^ RhoA GTPase and its downstream effector, ROCK kinase, are central regulators of actin cytoskeleton assembly and cellular tension.^51^ Mechanical stimulation rapidly activates the RhoA/ROCK pathway, promoting stress fiber formation and cellular contractility, which facilitates the transition of AT2 cells from a cuboidal to the flattened morphology of AT1 cells.^52^ This pathway is directly linked to YAP/TAZ activation, as ROCK-mediated cytoskeletal remodeling and nuclear deformation are key contributors to YAP/TAZ nuclear translocation.^53^ Treatment with the ROCK inhibitor Y27632 significantly suppresses stretch-induced AT1 differentiation and maintains AT2 marker expression.^53^^,^^54^

**Figure 2:**
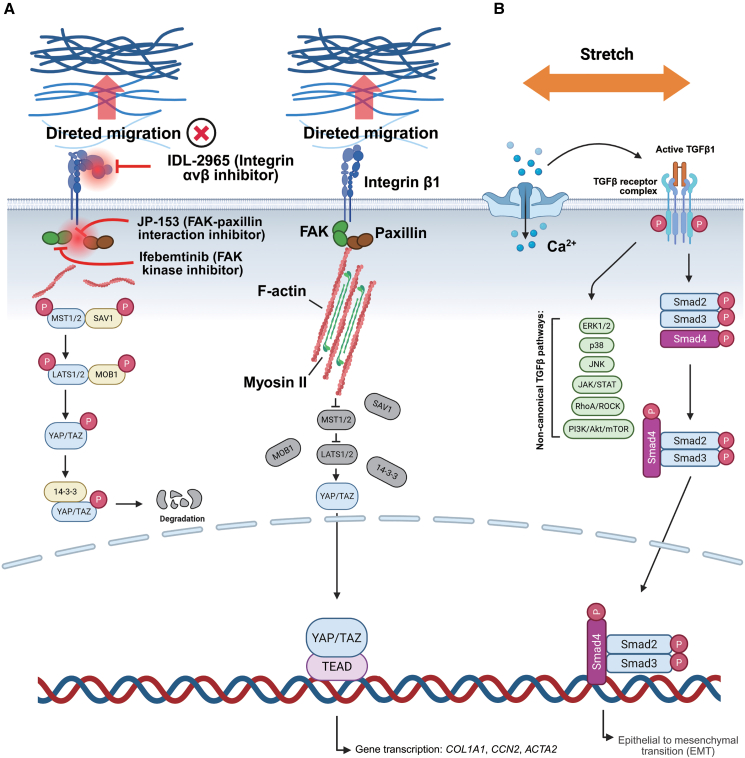
Abnormal mechanical microenvironments regulate multiple signaling pathways in alveolar epithelial cells to promote IPF progression (A) Increased ECM stiffness and altered topography guide durotactic cell migration through integrin-mediated focal adhesion signaling. Engagement of integrins (e.g., integrin β1) activates focal adhesion kinase (FAK) and paxillin, promoting actin cytoskeleton reorganization and myosin II-dependent contractility. Mechanical signal transmission along stressed actin fibers that inhibits YAP/TAZ phosphorylation, allowing their nuclear translocation and activation of profibrotic gene transcription, including *COL1A1, CCN2,* and *ACTA2*. Pharmacological inhibition of integrins and FAK disrupts focal adhesion maturation and impairs directional migration. (B) Cyclic mechanical stretch activates Piezo1 channels, leading to Ca^2+^ influx and activating TGF-β signaling, resulting in Smad2/3 phosphorylation and nuclear accumulation. This process promots epithelial-to-mesenchymal transition (EpMT) and fibroblast activation. This figure was created with BioRender software.

The mechanical stimuli experienced by alveolar AT2 cells primarily arise from both physiological and pathological forces. Under physiological conditions, the cyclic stretch generated by respiratory movements suggests the basic mechanical signal acting on the alveolar epithelium.^55^ During development, fetal breathing movements drive the aspiration of amniotic fluid, generating fluid shear stress and pressure that are critical for early alveolar development and AT1 cell differentiation.^56^^,^^57^ In pathological conditions, the alveolar structure is collapsed, accompanied by ECM stiffening, which results in AT2 cells being exposed to abnormal mechanical stimulation, disrupting their normal differentiation programs. More importantly, studies have shown that AT2 cells may undergo fundamental functional changes under pathological mechanical stress.^58^ For example, excessive activation of the Rho/ROCK pathway caused by abnormal mechanical stress can prevent AT2 cells from effectively differentiating into functional AT1 cells, thereby impairing alveolar repair.^8^^,^^59^ One classical mechanotransduction pathway involves the Piezo1 ion channel and the YAP signaling axis. Studies have shown that when cells are cultured on a stiff substrate or subjected to excessive stretch (e.g., 15% strain at 0.2 Hz simulating the breathing cycle), Piezo1 channels respond to membrane tension changes, opening to allow rapid Ca^2+^ influx. This process promotes downstream TGF-β secretion and triggers the nuclear translocation of YAP, activating the transcription of key fibrotic genes such as collagen I α1 (COL1A1) and connective tissue growth factor (CTGF)^60^ (Figure 2C). In a study by Garantziotis et al., AT2 cells were subjected to cyclic stretch using the Flexcell tension plus system. After four days of exposure to 15% stretch at 0.86 Hz, E-cadherin expression was significantly reduced, while vimentin levels were markedly upregulated, indicating that the cells had undergone EpMT^61^ (Figure 2C). This phenotypic shift not only compromises the epithelial barrier function of AT2 cells but also enables them to actively promote ECM synthesis and remodeling. As a result, AT2 cells contribute directly to lesion formation. Notably, YAP activation in this context is not transient; rather, it can be sustained through matrix stiffness-mediated positive feedback, functioning as a mechanical “amplifier” during the early stages of fibrosis.^62^

This finding reveals the transition of epithelial cells from “passive victims” to “active drivers” in fibrosis and suggests that the mechanical environment can serve as a target for early intervention.

### Fibroblast mechanical memory and epigenetic remodeling

Unlike epithelial cells, fibroblasts are the principal executors of ECM production. However, the traditional view of fibroblasts as passive responders to factors such as TGF-β no longer explains their persistent activation and resistance to reversal in IPF. Recent studies indicate that mechanical signals not only transiently activate fibroblasts but also imprint a “mechanical memory” at the chromatin level, altering epigenetic states that sustain high activation even after removal from stiff stimuli.

**Figure 3:**
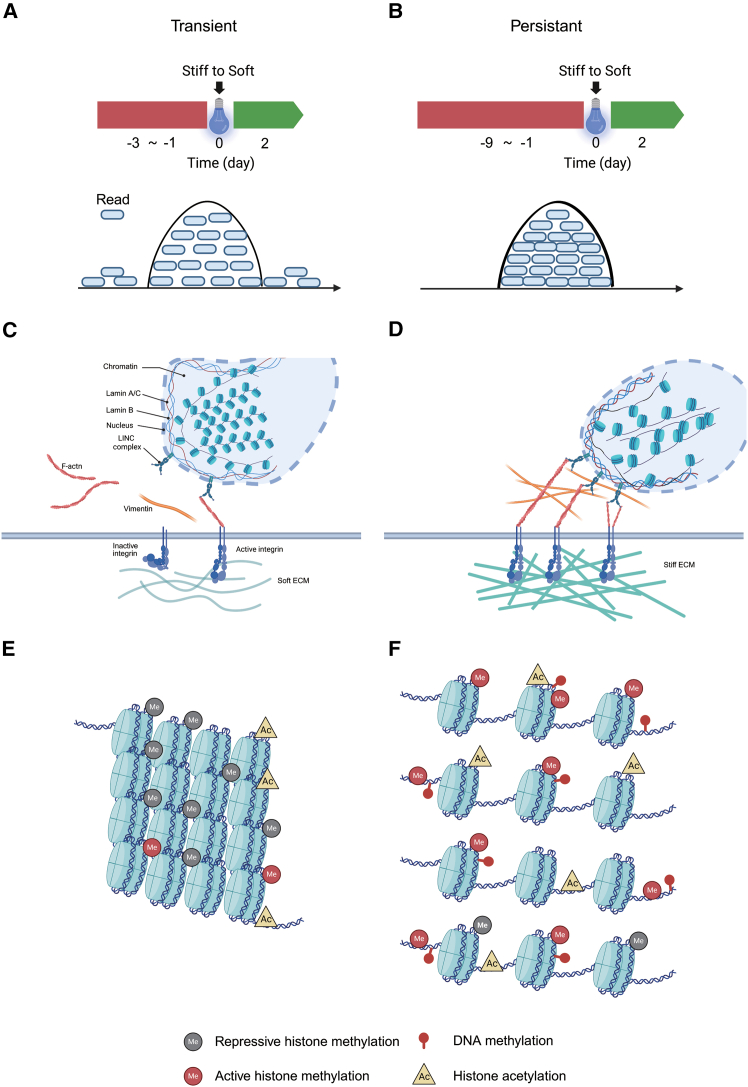
Mechanical signals promote the formation of mechanical memory in fibroblasts through epigenetic regulation (A and B) Lung fibroblasts cultured on tunable-stiffness hydrogel substrates, compared to those cultured short-term (3 days), fibroblasts cultured long-term (9 days) possess mechanical memory, indicating a sustained pro-fibrotic state. Mechanistic investigations further reveal that increased substrate stiffness induces chromatin condensation, leading to a global reduction of chromatin accessibility in fibroblasts, thereby promoting their transition to myofibroblasts. (C–F) On stiff ECM, mechanical signaling is initiated at the cell-ECM interface via integrins, leading to intracellular F-actin polymerization and contractility. This contraction stress is subsequently transmitted to the nucleus through the linker of nucleoskeleton and cytoskeleton (LINC) complex, where it regulates chromatin architecture by reducing chromatin compaction and increasing histone acetylation/methylation, ultimately promoting fibroblast activation. This figure was created with BioRender software.

Tschumperlin and colleagues have demonstrated that matrix stiffness plays a crucial role in fibroblast activation. When human lung fibroblasts were cultured on a stiff substrate (64 kPa) for 24 h, they significantly increased the expression of alpha-smooth muscle actin (α-SMA) and COL1A1, and maintained a persistent myofibroblast-like phenotype. Further mechanistic investigation revealed that this “mechanical memory” is associated with high levels of histone H3 lysine 9 methylation (H3K9me), which prevents fibroblasts from reverting to a quiescent state^63^ (Figures 3E and 3F). Notably, this memory effect appears to be reversible; fibroblasts exposed to a stiff substrate for 2–7 days and subsequently transferred to a soft substrate for 2 days showed a reduction in activation markers. Using ATAC-seq, genome-wide chromatin accessibility can be assessed, enabling the identification of regulatory DNA elements associated with transcriptional activity. The results revealed that transiently activated fibroblasts (3-day stiff) had higher chromatin accessibility compared to persistently activated fibroblasts (9-day stiff). This finding indicates that mechanical cues drive fibroblast activation in a time-dependent manner, in which short-term exposure to a stiff microenvironment preserves chromatin accessibility and transcriptional plasticity, while sustained mechanical loading induces epigenetic consolidation and long-term stabilization of profibrotic gene programs^64^ (Figures 3A–3D).

### Inflammatory fibroblasts as stiffness-responsive cells

In the pathological progression of IPF, increased stiffness of the alveolar ECM is not merely a consequence of the disease but also a key driver of its advancement.^65^ Activated fibroblasts and myofibroblasts within the pulmonary interstitium serve as the principal sensors and effectors of this pathological mechanical microenvironment.^66^ By sensing elevated ECM stiffness, these cells initiate and amplify a self-sustaining pathological signaling network centered on interleukin-6 (IL-6), ultimately leading to irreversible remodeling of lung architecture.^67^

Within a thickened and highly cross-linked ECM microenvironment, fibroblasts and myofibroblasts strengthen their adhesion to collagen and fibronectin through integrins such as αvβ6, thereby activating the FAK/SRC pathway and promoting nuclear translocation of the mechanosensitive co-activators YAP/TAZ.^68^^,^^69^ This mechanobiological signaling cascade induces the expression of multiple profibrotic mediators and regulators of matrix homeostasis, including IL-6, TGF-β1, COL1A1, and LOXL2, effectively converting mechanical stimuli into amplified biochemical signals. Because TGF-β signaling further enhances ECM deposition and cross-linking, this process establishes a bidirectional coupling between mechanical and biochemical cues, allowing increased matrix stiffness more to function as an “amplifier” during fibrotic progression rather than merely a passive outcome.^70^^,^^71^

IL-6 and its *trans*-signaling pathway (IL-6 binds to the soluble IL-6 receptor (sIL-6R) and subsequently signals through gp130) play a pivotal role within this amplification system. This mechanism bypasses the restricted expression of membrane-bound IL-6R, allowing IL-6 signaling to induce inflammation among multiple pulmonary cell types, including fibroblasts, AECs, and endothelial cells.^72^ Downstream pathways, including the JAK/STAT3, MAPK, and PI3K/AKT, not only interact with TGF-β/Smad but also promote stress fiber formation and α-SMA expression, which supports the survival and contractile function of activated myofibroblasts.^70^ These effects ultimately promote ECM deposition and further alter local mechanical properties. Some studies suggest that, under certain conditions, STAT3 can promote the accumulation of Smad3 in the nuclear and cooperatively mediate collagen gene transcription; however, the universality of these molecular mechanisms across different models remains to be fully validated.^73^ Beyond stromal cells, IL-6 signaling also contributes to the irreversible fibrosis of pulmonary tissue by acting on AECs.^74^ Sustained IL-6 activation may disturb AT2-to-AT1 differentiation and alveolar epithelial regeneration, while also inducing partial EpMT-like changes and an aberrant secretory phenotype in AT2 cells.^75^^,^^76^ Whether these epithelial alterations directly contribute to IPF remains controversial and requires further support from structural and lineage-tracing studies.

In terms of immune regulation, IL-6 induces the expression of chemokines such as CXCL1, CXCL8, and CCL2,^77^ thereby driving the recruitment of neutrophils and macrophages to the fibroblast foci, further promoting macrophage polarization toward a profibrotic M2-like phenotype. These immune cells, in turn, constitute major sources of TGF-β, platelet-derived growth factor (PDGF), and additional IL-6/sIL-6.^78^ In parallel, IL-6 facilitates Th17 cell differentiation while suppressing regulatory T cell (Treg) formation. In turn, IL-17A secreted by the Th17 cells can promote the proinflammatory and profibrotic phenotype of fibroblasts, establishing another positive feedback that is particularly prominent during the transition from inflammation to fibrosis.^79^

In summary, this section has systematically reviewed how macrophages, AECs, fibroblasts, and related cells sense and respond to mechanical stimuli, emphasizing the central role of mechanical signaling in inducing pro-fibrotic phenotypes. These findings provide an important biophysical basis for understanding the mechanically induced initiation of IPF.

## Tissue scale: ECM mechanical heterogeneity and the vicious cycle

While the molecular-level mechanosensing mechanism is recognized as the “microscopic engine” of IPF, tissue-scale mechanical heterogeneity constructs the “mechanical topography” that orchestrates disease progression. Within lung tissue, cells do not exist in isolation but are embedded in an ECM network composed of collagen, elastin, glycosaminoglycans, and various structural proteins.^62^^,^^64^ This network not only provides adhesion and structural support but also influences cell migration, differentiation, and fate decisions through its physical properties, such as stiffness, stress distribution, and microtopology.^80^ Remodeling of ECM increases spatial heterogeneity of tissue stiffness during the progression of IPF. Spatial heterogeneity of ECM stiffness creates spatially heterogeneous distributions of stresses during breathing. This causes mechanotransduction-induced fibroblast activation that further drives matrix remodeling. Therefore, stress stiffening and cellular remodeling couple into a biomechanical positive feedback loop where local stiffening, stress concentration, and cellular remodeling act synergistically to drive fibrosis forward.

### Mechanical heterogeneity and stress concentration at fibrotic lesion margins

A hallmark of IPF histopathology is the irregular, scattered distribution of fibroblastic foci within alveolar structures, accompanied by prominent matrix stiffening and structural collapse around these foci.^81^^,^^82^ Recent *in vivo* imaging and mechanical measurement studies have further revealed that these regions exhibit not only compositional changes but also pronounced physical heterogeneity. Such stiffness heterogeneity can drive fibroblasts to migrate directionally toward regions of higher rigidity, a process known as durotaxis. In a latest work, Al-Hilal et al. combined *in vivo* nanoindentation with fibrotic region labeling and demonstrated that fibrotic lung tissue displays steep and highly heterogeneous stiffness gradients, with local gradients reaching approximately 100–500 Pa·μm-1, markedly exceeding the relatively homogeneous mechanical properties of normal lung tissue. Importantly, this gradient range closely matches the threshold required to induce fibroblast durotactic migration^83^ (Figure 2A). Through genetic disruption (L994E FAK mutation) and pharmacological inhibition (JP-153), the authors further showed that the FAK-paxillin mechanosensing module is essential for durotaxis. Blocking this pathway significantly reduced fibroblast migration to stiff regions and attenuated lung fibrosis, suggesting that anti-durotactic strategies may have therapeutic potential^83^ (Figure 2A). Using micropatterned hydrogels composed of alternating 100 μm-wide “stiff” stripes and 200 μm-wide “soft” stripes, with elastic moduli of approximately 40 kPa and 4 kPa, respectively, a stiffness gradient of about 275 Pa μm^−1^ was generated, closely mimicking the spatial stiffness variations observed in fibrotic tissues *in vivo*.^84^ Fibroblasts exhibited directional migration along the stiffness gradient, independent of soluble chemotactic factors or matrix ligand gradients. Instead, migration was mediated through focal adhesion-cytoskeletal mechanobiological coupling, supporting durotaxis as an autonomous migration mode in fibrotic environments.^85^

In parallel, matrix stiffness itself can promote fibroblast phenotypic activation. Within tunable hydrogels ranging from 1 to 50 kPa, increasing stiffness significantly enhanced α-SMA expression, cytoskeletal remodeling, and migratory capacity of lung fibroblasts, and amplified their response to chemotactic cues such as PDGF. Silencing α-SMA attenuated these stiffness-dependent migratory effects.^86^ Furthermore, primary human lung fibroblasts cultured on stiffness-gradient hydrogels were shown to sense increasing matrix rigidity through alterations in mitochondrial dynamics. This process involved DRP1/MFF-dependent mitochondrial fission, increased ATP production, and polarized mitochondrial localization toward the leading edge to meet the elevated energy demand of durotactic migration. The activation of the DRP1/MFF pathway was observed in both IPF patient samples and bleomycin-induced fibrosis models, linking this metabolic-mechanical axis to disease progression.^87^ These cellular responses to matrix stiffness are closely associated with tissue-level mechanical remodeling during IPF progression. 3D-UTE MRI enables three-dimensional imaging of collagen-rich, densely structured, or highly fibrotic tissues by acquiring signals at ultrashort echo times. Using this approach, deformation mapping in IPF lungs demonstrated significantly reduced tissue deformability compared with healthy controls, indicated by a lower mean Jacobian determinant (Jac-mean; 0.21 ± 0.08 vs. 0.27 ± 0.07, *p* < 0.001). Young’s modulus was found to be over three times higher than in controls.^88^^,^^89^ Atomic force microscopy (AFM) analysis by Davies et al. demonstrated that, while collagen content is markedly elevated in IPF lung tissue, the increase in tissue stiffness was more closely associated with enhanced collagen crosslinking and ECM microstructural remodeling than with collagen abundance alone.^90^

Finite element modeling, a computational approach that discretizes tissues into small elements to simulate mechanical behavior under defined stiffness gradients, has shown that when matrix stiffness increases from 3 kPa to 65 kPa over a 1 mm scale, fibroblast migration velocity increases by more than 40%.^91^ Notably, within a local range of 0–160 μm, cells maintained a high degree of directional persistence, migrating predominantly within a ±30° angle relative to the gradient, indicating that stiffness gradients enhance both migration efficiency and directional guidance toward stiffer regions. These findings were observed in 2D experimental systems and may not fully reflect responses in 3D *in vivo* environments.^92^ This suggests that fibrotic lesions are not static endpoints but dynamic “mechanical attractors” that recruit and activate surrounding cells. Indeed, in IPF tissues, fibrotic lesions often display a radial or “waterfall-like” organization, where collagen fibers are aligned along the axes of airways or interlobular septa. This distinct spatial pattern reflects the underlying diffusion of mechanical signals during disease progression.^12^^,^^93^

Crucially, this mechanical transmission exhibits spatial selectivity. High-resolution (HRCT) imaging reveals fibrosis preferentially expands along the lung lobar inferior margins and subpleural zones, regions that are structurally fragile and subject to elevated tensile stress (see Section 4).^15^^,^^94^ Hence, ECM heterogeneity is not merely a consequence of fibrosis but a determinant of its propagation pathways.

### ECM stiffness-driven regulation of fibroblast survival, apoptotic sensitivity, and aging

ECM stiffening not only provides persistent mechanical activation signals but also reshapes the metabolic program of myofibroblasts. Increased matrix stiffness promotes the persistence of myofibroblast activation and survival through the integrin-FAK-YAP/TAZ axis. These signals effectively render myofibroblasts addicted to persistent mechanochemical support and pro-survival signaling.^37^ Stiff ECM reprograms cellular energy metabolism by shifting mitochondrial dynamics from fusion toward fission through the DRP1/MFF-dependent pathway, which leads to mitochondrial network fragmentation, enhanced oxidative phosphorylation, increased ATP production, and elevated ROS levels.^95^ This metabolic adaptation provides the energetic requirement for long-term survival of myofibroblasts in stiffened ECM. However, accumulating evidence indicates that sustained myofibroblast activation does not confer true apoptosis resistance. Instead, these cells maintain a state of increased mitochondrial apoptotic priming. Under the combined mechanical and metabolic stress imposed by a stiff ECM, the balance of BCL-2 family proteins is reset, with the upregulation of pro-apoptotic factors accompanied by compensatory increases in pro-survival proteins such as BCL-XL, rendering cell survival critically dependent on the latter to maintain mitochondrial outer membrane integrity.^34^ This “addiction-like survival” represents a central consequence of ECM stiffening: Although myofibroblast survival appears enhanced, the intrinsic apoptotic threshold is in fact lowered. Mechanistically, inhibition of key mechanotransduction nodes (integrins, FAK, ROCK, or YAP) or blockade of survival proteins such as BCL-XL on stiff substrates rapidly triggers mitochondrial outer membrane permeabilization (MOMP) and selective apoptosis. Consistent with this primed state, myofibroblasts cultured on stiff matrices show high sensitivity to BH3 mimetics (e.g., ABT-263) and mechanotransduction inhibitors, whereas such effects are absent in cells on soft matrices or in quiescent fibroblasts.^37^

Notably, ECM stiffening does not exclusively predispose fibroblasts to apoptosis; under sustained mechanical stress, it can also drive fibroblasts toward a senescent fate. For instance, stiff ECM also promotes abnormal epithelial-mesenchymal interactions, a process mediated at least in part by senescence-associated secretory phenotype (SASP) factors.^66^ Senescent cells can influence neighboring cells through SASP, giving rise to the so-called senescence-induced bystander effect.^96^ This process may drive adjacent cells into a senescent state, thereby sustaining aberrant wound repair responses even in the absence of ongoing external injury.^82^^,^^88^ In addition, the ECM is a dynamic system in which most ECM proteins interact with structural cells to regulate intracellular signaling pathways and promote the production of SASP factors. For example, collagen can induce cellular senescence through integrin/Akt signaling.^97^ Decorin promotes cell-cycle arrest by upregulating p16, p21, and phosphorylated p53 via CCN1/CCN2-mediated RAS/RAF and RAC1/ROS pathways.^98^^,^^99^ Elastin and fibulin enhance senescence programs through PPARγ- and Wnt/β-catenin/ROS-dependent signaling.^100^^,^^101^

### Role of ECM viscoelasticity in IPF progression

However, tissue mechanics are not determined by stiffness alone; the fibrotic ECM also displays viscoelastic properties that may influence how cells respond to mechanical cues. Current understanding of viscoelastic alterations in IPF is still developing. Viscoelasticity describes the combined viscous (energy dissipation) and elastic (energy storage) behavior of materials and is essential for efficient lung expansion and recoil in healthy tissue.^102^ In IPF, however, this balance gives rise to a mechanical “paradox.” While excessive collagen deposition increases the overall elastic modulus of the tissue, parameters such as stress relaxation, tan δ (the viscous-to-elastic ratio), and relaxation time constants do not decrease linearly with increasing stiffness. Instead, these viscoelastic features are partially retained or remodeled.^103^^,^^104^ This mechanical signature indicates that the ECM evolves into a heterogeneous and dynamic mechanical microenvironment during fibrosis, where stress concentration and time-dependent mechanical loading influence cellular behavior. For example, matrices with higher viscous dissipation have been shown to promote stronger stress fiber formation in fibroblasts, increase α-SMA and collagen expression, and enhance cellular sensitivity to external mechanical cues, thereby supporting the sustained activation of inflammatory and fibrotic signaling pathways.^105^ Consistent with this complexity, the viscoelastic behavior of IPF ECM often requires multi-element Maxwell models (e.g., four or more elements) for accurate fitting, with higher relative weighting factors (Ri) than those observed in healthy tissue, highlighting the prominence of time-dependent mechanical responses in fibrotic ECM.^89^

To capture these features, biomimetic models such as hydrogel systems, including hyaluronic acid-based and decellularized ECM hydrogels, have been employed to recapitulate the mechanical environments of healthy and fibrotic lungs and to investigate how mechanical stress contributes to lesion distribution and fibrosis progression.^103^^,^^105^ Overall, IPF should not be viewed as a condition of ECM stiffening alone, but rather as a combined process of increased stiffness and viscoelastic remodeling. A deeper understanding of these mechanical changes may provide additional perspectives for the development of therapeutic strategies targeting pulmonary fibrosis.

### Hemodynamic disturbances in perivascular niches and endothelial mesenchymal transformation (EnMT)

At another critical tissue scale dimension, the perivascular microenvironment experiences fluid mechanical perturbations. IPF features not only parenchymal alveolar fibrosis but also vascular remodeling and microvascular rarefaction.^106^^,^^107^ The perivascular “niche,” anatomically adjacent to fibroblastic foci, represents a highly active biomechanical-biological coupling zone.^108^^,^^109^ Increasing evidence indicates that shear stress regulates the fate of pulmonary microvascular endothelial cells (PMVECs).^110^ Microfluidic chip studies simulating pulmonary vascular shear stress demonstrate that prolonged exposure of PMVECs to 4 dyn/cm^2^ shear stress reduces endothelial markers (e.g., VE-cadherin and CD31) and upregulates EnMT markers such as Snail and Vimentin, indicating phenotypic drift toward fibroblast-like cells.^111^ Hypoxia synergizes with fluid mechanical stress to activate the HIF-1α signaling axis, promoting aberrant vascular endothelial growth factor (VEGF) secretion, which exacerbates local vascular permeability and tissue edema, further disrupting mechanical homeostasis.^112^ In animal models, inhibition of EnMT pathways significantly reduces pulmonary collagen deposition and tissue stiffening, implicating EnMT as a bridging mechanism between hemodynamic abnormalities and fibrosis.^113^^,^^114^^,^^115^ These findings indicate that a stiff ECM functions not only as a supportive niche for myofibroblast survival but also as a mechanical context that locks cells into a fragile equilibrium, rendering them highly vulnerable to targeted disruption.

**Figure 4:**
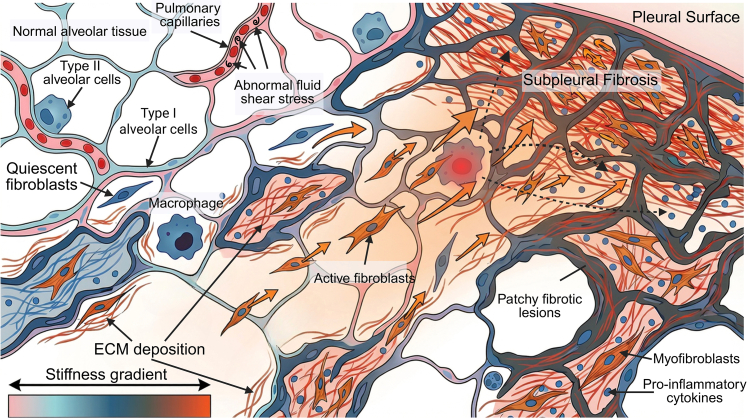
Formation of spatial stiffness heterogeneity in alveolar tissue and its impact on IPF progression Localized aberrant stress may cause epithelial injury, leading to abnormal ECM deposition and increased tissue stiffness. In addition, occlusion of peripheral capillaries can generate abnormal shear stress, resulting in vascular stiffening and reduced gas exchange capacity. The elevated ECM stiffness at lesion areas can drive the surrounding fibroblasts to migrate toward the stiffer (fibrotic) areas and secrete more ECM, which accelerates the progression of fibrosis. On the other hand, resident macrophages can respond to abnormal mechanical stress and tissue remodeling by secreting pro-inflammatory cytokines, which further promote fibroblast activation and matrix deposition. This positive feedback between ECM stiffening, fibroblast migration, and pro-inflammatory macrophage activation contributes to the spatial expansion of fibrosis in IPF. This figure was created with BioRender software.

In summary, the mechanical environment at the tissue scale in IPF is highly dynamic and heterogeneous. From strain concentration at fibrotic lesion edges to shear stress disturbances in vascular niches, each microdomain constitutes a local “mechanical stress source.” These stresses in turn regulate cell migration, activation, and secretion, intensifying ECM remodeling and stiffness, forming a classical mechanical positive feedback loop (Figure 4). This section focuses on ECM remodeling and mechanical heterogeneity at the tissue scale. It explains how local changes in the mechanical environment amplify cellular mechanosensing through stress concentration and force transmission, thereby driving the formation and expansion of fibrotic foci.

## Organ scale: regional specificity of respiratory biomechanics

IPF is a disease characterized by marked spatial heterogeneity, with its most clinically and radiologically recognized feature being a distinct “regional predilection” - fibrosis typically initiates at the lung bases, subpleural regions, and posterior basal segments, then progressively extends throughout the lung parenchyma.^13^^,^^116^^,^^117^ Traditional explanations have largely relied on local anatomical differences, perfusion patterns, or oxidative stress states.^118^^,^^119^^,^^120^ Recently, however, organ-scale respiratory biomechanics—specifically the regional distribution of stresses and strains experienced by the lung during breathing—have emerged as a core physical driving mechanism underlying this regional pathology.^121^

### Why are the lung bases more “susceptible”? Insights from respiratory mechanics

In healthy individuals, lung expansion during a quiet breathing cycle is not uniform across regions. Imaging studies have demonstrated that during inspiration, strain amplitudes at the lung bases reach 12–15%, while those at the apex often remain below 8%. This regional disparity arises from a combination of physical and physiological factors.^96^^,^^122^

• Gravitational gradients and vertical tissue compression: In standing or sitting posture, gravity induces an intrapulmonary pressure gradient that places the basal lung at a lower functional residual capacity (FRC) at end-expiration, thereby enabling greater expansion potential during inspiration.^123^
• Diaphragm-driven preferential transmission to lung bases: As the primary inspiratory muscle, the diaphragm’s contraction primarily displaces lower lobe lung tissue ventrally, producing a “strain amplification effect.”^124^
• Enhanced pleural traction at lung bases: The subpleural regions at the lung bases experience the greatest mechanical pulling during the respiratory cycle, resulting in higher amplitude mechanical fluctuations.^125^

In patients with IPF, rather than diminishing, these regional strain differences are amplified. Functional imaging modalities such as dynamic HRCT and ventilation MRI confirm that, despite overall reduced lung compliance, “excessive strain” at the lung bases persists in early disease stages, with strain amplitudes exceeding normal values by more than 30%. These “residual stresses” or “micro-areas of overstrain” are considered biomechanical niches that trigger fibrotic lesion formation.^15^^,^^126^^,^^127^

### How breathing mechanics drive fibrosis propagation along structural pathways

Mechanical tension generated by breathing is not confined locally but propagates as stress waves along the pulmonary Interstitium. The ECM network within lung tissue acts as a heterogeneous continuous medium, whose structural connectivity facilitates “distal mechanical coupling” between alveoli via matrix fibers.^39^^,^^128^^,^^129^ When ECM thickening and stiffening first occur in a certain basal region, the surrounding stress distribution inevitably alters, creating a “mechanical tension island” centered on the lesion. This biomechanical niche attracts fibroblast migration and activation, thereby promoting fibrosis progression along the lobar axis.^86^

Clinical observations confirm this propagation pattern: Fibrosis initially manifests along the basal pleura on HRCT images, then advances centrally along bronchovascular bundles.^130^ Finite element simulations further demonstrate that localized stiffness perturbations in lung tissue (e.g., a 1–2 kPa increase in initial lesion stiffness) significantly reshape stress concentration zones within a 5–10 mm radius, providing a “physical frontier” for fibrotic expansion.^126^^,^^131^

### Respiratory mechanics under clinical intervention: The double-edged sword of mechanical ventilation

Notably, mechanical ventilation as a vital life-support intervention can itself induce or exacerbate pulmonary fibrosis, presenting a risk of “iatrogenic mechanical injury.” Retrospective studies in patients with ARDS show that ventilation with high tidal volumes (>10 mL/kg) increases the risk of developing IPF-like features by 2.3-fold. Protective ventilation strategies, involving lower tidal volumes and appropriate positive end-expiratory pressure (PEEP, 4–8 mL/kg), effectively reduce pathological lung stress concentration and are recognized as key in preventing “mechanical ventilation-induced fibrosis.”^132^^,^^133^ Animal experiments also confirm that applying high-pressure ventilation in healthy rats induces ECM deposition and TGF-β upregulation specifically at lung bases, indicating that purely mechanical stimuli can activate fibrotic pathways.^134^

### Prediction of mechanical “hotspots” and prospects for personalized intervention

With advances in lung imaging and computational modeling, organ-scale stress distributions can now be reconstructed using patient-specific 3D lung models.^135^ By reconstructing lung structures from CT data and simulating respiratory-driven strain responses, potential “mechanical hotspots” can be identified.^136^ Animal studies and *in vitro* engineering research suggest that such regions of elevated mechanical stress may contribute to localized fibrosis progression, and targeted modulation of ECM properties could potentially influence disease outcomes.^137^

In particular, ECM-softening material injections have been explored in animal models and preclinical engineering studies as a strategy to locally reduce tissue stiffness and modulate fibroblast activity. These studies indicate that controlled softening of fibrotic regions may mitigate ECM accumulation and influence cell behavior.^138^ However, it is important to note that this approach remains at an experimental stage and has not yet been established as a clinically approved therapy. Its safety, efficacy, and optimal delivery strategies in humans remain to be determined.

Consequently, while the identification of mechanical hotspots offers promising avenues for personalized intervention, current applications of ECM-softening strategies are largely theoretical or preclinical. Future work combining patient-specific modeling with experimental validation may guide the development of targeted therapies, optimize intervention strategies, and inform clinical decision-making, including surgical planning and individualized rehabilitation protocols.

This section reviews organ-scale dynamics, focusing on strain distributions generated by breathing and regional mechanical differences, which provide key insights into the spatially selective development of pulmonary fibrosis. Studying mechanics at this scale allows local tissue changes to be interpreted within the context of overall lung function.

## Therapeutic strategies targeting mechanical pathways

Following the multiscale mechanistic analyses described above, recent studies have increasingly integrated mechanobiological knowledge into strategies to intervene in IPF. Unlike the previous sections, which focused on disease initiation and progression, this section highlights research directly relevant to therapeutic and translational applications, including mechanoregulation-based pharmacological interventions, biomaterial strategies, and emerging frameworks for mechanically guided therapy. It should be noted that some contents of the discussion are based on experimental and preclinical studies, which aim to summarize current mechanobiology-based intervention strategies rather than established clinical treatments.

### Reinterpreting mechanical mechanisms in anti-fibrotic drugs

The two representative drugs currently approved for IPF treatment are Nintedanib and Pirfenidone, although traditionally attributed to targeting growth factor pathways or inflammation regulation, recent studies suggest that their efficacy may also partly rely on modulating the cell-ECM mechanical interface.

Several studies have shown that nintedanib can reduce fibroblast contractility and actin stress fiber formation by inhibiting tyrosine kinase signaling pathways involving fibroblast growth factor (FGF), VEGF, and PDGF.^139^^,^^140^ In an IPF-derived ECM culture model, normal fibroblasts differentiated toward a myofibroblast-like phenotype. Treatment with nintedanib (100 nmol/L) led to a significant reduction in cell area and cell aggregation, indicating a direct effect in lowering cellular tensile force and tissue contractility.^141^

Pirfenidone is a small-molecule drug with multiple biological regulatory functions. It has been reported to attenuate fibroblast-mediated collagen matrix contraction and reduce ECM stiffness by downregulating α-SMA expression in myofibroblasts.^142^ In engineered 3D tissue models, pirfenidone was shown to decrease both tissue contractile force and elastic stiffness. Additionally, it interferes with the MRTF-actin axis, thereby suppressing mechanically induced transcriptional responses.^143^

In addition to pirfenidone and nintedanib, nerandomilast has recently been approved as a novel antifibrotic agent for the treatment of IPF, representing an important advance in therapeutically targeting the inflammation-fibrosis coupling axis. Nerandomilast is an orally available, selective phosphodiesterase-4 (PDE4) inhibitor. By increasing intracellular cyclic adenosine monophosphate (cAMP) levels, it suppresses the activation of multiple pro-inflammatory signaling pathways, thereby attenuating fibroblast activation, myofibroblast differentiation, and excessive ECM deposition.^144^^,^^145^ Early phase II clinical trials demonstrated that treatment with nerandomilast significantly slowed the annual rate of decline in forced vital capacity (FVC), with lung function being stabilized or modestly improved in a subset of patients.^146^ Subsequent pivotal clinical studies further confirmed its antifibrotic efficacy. Compared with placebo, nerandomilast was shown to reduce disease progression in patients with IPF who were either treatment-naïve or receiving background standard antifibrotic therapy, suggesting that it may be effective both as monotherapy and as a potential add-on to existing treatments.^146^ With respect to safety, clinical trial data indicate that nerandomilast is generally well tolerated. Diarrhea was the most commonly reported adverse event, while the incidence of serious adverse events remained low, with no significant increase in treatment-related safety risks.^146^ Collectively, these findings support nerandomilast as a promising next-generation antifibrotic agent and highlight its emerging clinical value in the evolving therapeutic landscape of IPF.

While these drugs can slow disease progression in clinical settings, their ability to reverse established fibrotic regions with stiff ECM appears limited. This suggests that interventions targeting the mechanobiological feedback underlying fibrosis may be most effective during early disease stages, before irreversible matrix remodeling occurs. In other words, beyond targeting cytokines and inflammation, strategies that directly modulate the mechanical microenvironment of fibrotic tissue may be essential for promoting fibrosis resolution. Based on these observations, current strategies aimed at targeting mechanical feedback in pulmonary fibrosis can be broadly classified into three approaches.

First, reducing matrix stiffness by inhibiting collagen deposition or decreasing collagen crosslinking can allow fibroblasts to re-experience a more “physiological” mechanical environment, thereby lowering their propensity for myofibroblast differentiation.^147^

Second, pharmacological or genetic interventions targeting mechanotransduction pathways, including integrins, FAK/ROCK, and Piezo1/2, can interrupt pathological mechanical signaling. Integrins serve as key receptors through which fibroblasts sense ECM mechanics, providing a structural basis for mechanotransduction.^148^ Downstream, the FAK-paxillin complex within focal adhesions functions as a core mechanosensor, integrating cellular traction forces and regulating adhesion dynamics and migration.^149^ This mechanosensor mediates directional fibroblast migration along stiffness gradients, promoting the accumulation of fibroblasts in progressively stiffened fibrotic regions and accelerating fibrosis propagation.^150^ Mechanical signals transmitted via FAK-paxillin are further amplified through cytoskeletal tension pathways, enhancing RhoA/ROCK-dependent contractility, stress fiber formation, and nuclear translocation of mechanosensitive transcription factors such as YAP/TAZ and MRTF, which drive sustained expression of pro-fibrotic genes including α-SMA and collagen.^150^ Notably, these pathways are also required for epithelial and endothelial repair in the lung,^151^ highlighting the challenge of selectively targeting pathological mechanotransduction without impairing normal tissue function.

Third, enhancing ECM degradation by activating matrix metalloproteinases (MMPs) or improving matrix turnover may reduce stress concentration and mechanical barrier effects within fibrotic tissue. Such strategies likewise require precise spatial control, as excessive MMP activity, particularly MMP-9, can damage the basement membrane and accelerate disease progression.^152^

In summary, fibrosis resolution is not simply the cessation of inflammatory or immune responses, but rather a process involving a mechanical “reset” of the microenvironment. Mechanobiology provides a novel conceptual framework for studying fibrosis resolution: By remodeling or redefining the mechanical context experienced by cells, fibrotic cells can be guided back toward homeostasis or directed into apoptosis/deactivation, ultimately achieving structural and functional repair.

### Matrix engineering and biomaterials: Advanced approaches to flexible microenvironment remodeling

In recent years, the intersection of tissue engineering and materials science has offered novel perspectives for mechanically targeted therapy. By designing biomaterials capable of “mechanical microenvironment reprogramming,” researchers seek to physically reconstruct the lung microenvironment’s mechanical properties, focusing on “softening” rather than merely “inhibiting,” thus inaugurating a new era of “mechano-therapeutics.”

For example, decellularized hydrogels derived from porcine lungs, administered at 1 mg/mL and 2 mg/mL in bleomycin-induced rat pulmonary fibrosis models, alleviated lung inflammation, oxidative damage, and fibrosis-associated protein markers such as α-SMA and type I collagen, demonstrating the potential of ECM-derived hydrogels as early-stage interventions to halt fibrosis progression.^138^ More advanced strategies combine drug delivery with mechanical regulation, such as co-loading the YAP inhibitor verteporfin and pirfenidone into targeted nanoparticles that accumulate extensively in lung tissue via aerosol inhalation. Released verteporfin inhibits airway epithelial fluidization and cyst formation, while pirfenidone suppresses fibroblast overactivation and reduces cytokine secretion, promoting epithelial fluidization, effectively improving lung function.^153^ This material-based intervention offers advantages of high target specificity, minimal side effects, and local controllability, showing promising translational potential amid the frequent systemic adverse effects of current anti-fibrotic drugs.

### Mechanical optimization strategies in respiratory rehabilitation: from training techniques to targeted sites

Alongside biomaterials, a more widely applicable intervention direction is rehabilitation training optimization based on respiratory mechanics analysis. Traditional respiratory training has largely focused on lung capacity and ventilation efficiency. Recent studies indicate that rationally designed breathing patterns can not only improve oxygenation but also modulate regional pulmonary stress distribution and slow the formation of “mechanical hotspots.”^154^^,^^155^^,^^156^

Diaphragm training and deep slow breathing have been demonstrated to significantly reduce basal region strain amplitude and pleural traction. Ultrasound strain imaging shows that diaphragm training can reduce average lower lobe strain by approximately 20%.^157^^,^^158^^,^^159^ In AI-assisted rehabilitation systems, respiratory feedback control devices have been developed to monitor lung expansion patterns in real time and adjust inspiratory depth and rate, enabling personalized “stress management.”^160^ Some groups also combine breathing exercises with Galileo vibration plate to synergistically regulate ECM homeostasis via micro-vibrations (6–20 Hz), constructing novel rehabilitation pathways integrating physical, neural, and biochemical modalities. These strategies are noninvasive, low-cost, and show high patient compliance, making them highly valuable for long-term IPF chronic disease management.^161^

In summary, therapeutic strategies targeting mechanical pathways are no longer limited to the regulation of molecular pathways but have evolved toward diversified physical interventions and tissue microenvironment remodeling. From the latent mechanical regulatory mechanisms in anti-fibrotic drugs, through responsive and adaptable biomaterial platforms, to rehabilitation programs based on respiratory mechanics, a more engineering-driven and multiscale treatment system is emerging. Future integration of imaging-guided mechanical assessment, AI-driven intervention feedback, and individualized physiological regulation will be essential for bringing mechanical therapy truly “bedside.”

## Challenges and prospects of multiscale integrative research

The development of IPF involves coordinated processes across spatiotemporal scales, which range from molecular signaling and cellular behavior to microenvironmental mechanics and organ-level remodeling. Understanding its progression and developing effective interventions requires an integrative research approach that bridges molecular mechanisms, cell matrix dynamics, and organ-level remodeling.

### Challenges in integrating biological and mechanical multiscale models

Despite recent advances in multiscale modeling approaches, such as cellular automata, finite element methods, and agent-based modeling, which have been widely applied to simulate lung structure and pathological evolution, effectively coupling biological signaling pathways with mechanical response models still faces significant technical and theoretical challenges.^162^^,^^163^ However, several challenges remain. First, temporal and spatial scale mismatches exist: Molecular signaling changes occur on the order of seconds or subcellular scales, whereas tissue remodeling takes days or weeks, requiring fundamentally different mathematical formulations and computational frameworks.^161^ Second, high parameter dimensionality is an issue: The heterogeneous nature of real lung tissue often requires hundreds of parameters (e.g., local stiffness, viscoelasticity, and strain rates), which can lead to a “curse of dimensionality” during model training and optimization.^162^^,^^163^ Third, scarcity of validation data limits progress: *In vivo* lung imaging technologies that provide both high spatiotemporal resolution and mechanical measurements are lacking, hindering closed-loop validation between real and virtual systems.^164^^,^^165^

A fundamental change is required to overcome current limitations in modeling complex biological systems. This involves establishing a framework that integrates multimodal data across spatial and temporal scales and couples structural patterns with functional outcomes. Notably, the application of machine learning and physics-informed neural networks (PINNs) enables the development of hybrid models that synergize data-driven inference with physiologically constrained dynamics, offering both predictive power and mechanistic interpretability.^164^^,^^165^

### Limitations of 2D models for studying 3D mechanosensing

Although two-dimensional (2D) culture systems have been widely used to investigate cellular mechanosensing mechanisms, fundamental differences exist between 2D and three-dimensional (3D) microenvironments. In 2D systems, cells adhere to a flat and mechanically uniform substrate, where force transmission is largely restricted to the basal surface through focal adhesions. This artificial geometry constrains cell morphology and polarity, and can amplify stiffness-dependent signaling responses compared with physiological tissue environments.^166^

In contrast, 3D matrices provide spatially distributed mechanical cues, in which cells are embedded within an ECM network and experience mechanical resistance, confinement, and force transmission in all directions. Under these conditions, cellular mechanotransduction is regulated not only by bulk stiffness, but also by ECM architecture, fiber organization, viscoelasticity, and matrix degradability, all of which are absent or poorly represented in 2D systems.^167^ For instance, Matera et al. revealed that myofibroblast differentiation in 2D was primarily dependent on matrix stiffness, whereas in 3D it was largely independent of matrix stiffness but correlated with matrix fibers. Further mechanistic studies demonstrated that matrix metalloproteinase activity was essential for myofibroblast differentiation in 3D.^167^

Importantly, several studies have demonstrated that mechanosensitive pathways identified in 2D cultures, including integrin-mediated signaling and YAP/TAZ activation, can exhibit altered activation thresholds, temporal dynamics, or even qualitatively different responses when examined in 3D environments.^168^ As a result, direct translation of stiffness-dependent findings from 2D experiments to 3D tissue contexts remains challenging and incompletely understood.

### Precise phenotyping and subgroup mechanical feature extraction under phenotypic heterogeneity

Clinically, patients with IPF exhibit significant heterogeneity in onset age, progression speed, and therapeutic response. This phenotypic heterogeneity poses major challenges for existing research. Traditional mechanics analyses, which focus on averaged values, fail to capture key clues arising from individual differences. To address this, future studies should emphasize several directions. First, establishing biomechanical biomarker lineages could provide new dimensions for patient stratification. Examples include local pulmonary strain distribution patterns, airway stretch frequencies, and mechanosensitive fibroblast migration trajectories.^96^^,^^166^ Second, integrating radiomics with biomechanical parameter modeling may correlate quantifiable texture features from HRCT images with regional stiffness distributions, helping to identify high-risk subgroups.^169^ Third, constructing a “biomechanics-molecular joint phenotyping atlas” by combining transcriptomic data, single-cell mechanotransduction pathway expression, and lung stress field simulations could reveal key pathological cross-nodes.^170^ These approaches are expected to drive the precision medicine transition from disease diagnosis toward subgroup prediction and from population-level analysis to individualized modeling.

### Bottlenecks in data integration and measurement technology development

Multiscale research urgently requires multisource data, yet the acquisition and integration of pulmonary mechanical data face several challenges: Precise measurement of tissue mechanics remains limited, noninvasive, *in vivo*, spatially resolved lung stiffness imaging technologies are lacking. Although magnetic resonance elastography (MRE) shows potential, its resolution is compromised by pulmonary gas interference^98^^,^^99^; *in situ* observation of cellular mechanosensing mechanisms is insufficient-most existing methods rely on 2D stretch/shear devices that poorly replicate alveolar 3D mechanical microenvironments^171^^,^^172^; synchronous force-ventilation data during breathing are rare-even when lung volume changes are recorded, local stress-strain coupling data are typically unavailable, limiting the precise definition of model boundary conditions.^101^

Therefore, the development of high-resolution alveolar-scale micro-elastic imaging techniques, *in situ* stretch-based cellular force spectrum measurement platforms, and combined imaging-simulation ventilation mechanics fusion technologies will provide solid support for future cross-scale research.

### The mechanistic coupling between mechanics and biological signals remains to be elucidated

Mechanical changes are not merely consequences of disease but actively contribute to bio-signaling activation. Key pathways such as YAP/TAZ, TGF-β, and FAK are significantly upregulated in alveolar cells exposed to sustained tension, promoting fibroblast phenotypic transitions.^100^^,^^173^^,^^174^ Strain-induced regulation of ECM synthesis has also been demonstrated: Tensile stimulation upregulates COL1A1 and FN1 gene expression, directly increasing matrix stiffness and forming a positive feedback loop.^175^^,^^176^ Moreover, mechanical fluctuations modulate immune activation; for example, one study found that fibroblasts co-cultured with M2 macrophages significantly enhanced fibroblast activation, even on soft substrates, highlighting the complex regulatory interplay among macrophages, matrix stiffness, and fibroblasts.^177^ Similarly, another study co-cultured macrophages and fibroblasts within engineered micro-lung tissues and found that M2 macrophages promoted extensive fibrotic remodeling in these constructs. Treatment with pirfenidone markedly reduced fibrosis, and mechanistic investigations revealed that pirfenidone suppressed macrophage mechano-activation via the inhibition of ROCK2 and integrin αMβ2.^178^ However, most current studies rely on a single cell type or 2D models. Future research should aim to develop integrated platforms that combine alveolar organoids, dynamic mechanical stimulation, and spatiotemporally resolved omics analyses to uncover the complex coupling between mechanics, signaling, and cellular behavior.

This review systematically examines the pathogenesis and progression of IPF from a multiscale mechanobiology perspective, highlighting that IPF should no longer be viewed merely as a disorder of inflammation or cytokine dysregulation, but as a complex multiscale system driven by mechano-biological feedback. Mechanical microenvironments not only shape the spatial distribution and dynamics of fibrosis but also offer operable targets for early intervention. In the future, integrating mechanobiology with high-resolution imaging, artificial intelligence modeling, and spatial medicine holds promise for precise prediction, mechanistic insight, and individualized therapy of IPF. This multiscale integrative approach not only deepens our understanding of pulmonary fibrosis but also establishes a new paradigm for the study and clinical translation of chronic fibrotic diseases.

## Acknowledgments

This work was supported by the 10.13039/501100001809National Natural Science Foundation of China (32201115), the Suzhou Industrial Park Healthcare Talent Support Initiative (2024) 31, and the Suzhou Municipal Applied Basic Research (Medical and Health) Youth Program (SYW2024168). A Project Funded by the 10.13039/501100012246Priority Academic Program Development of Jiangsu Higher Education Institutions (PAPD), the 10.13039/501100004608Natural Science Foundation of Jiangsu Province, China (BK20201183), the “innovative and entrepreneurial talent” in Jiangsu Province (JSSCRC2021568), and the “distinguished medical expert” in Jiangsu Province (JSTPYXZJ2021006).

## Author contributions

All authors participated in the collection and screening of relevant literature. R.Z. and T.X. wrote the original draft. R.Z., T.X., and Z.Y. revised the manuscript. R.Z., T.X., and Z.Y. supported funding of the work.

## Declaration of interests

The authors declare no competing interests.

## Declaration of generative AI and AI-assisted technologies in the writing process

During the preparation of this manuscript, the authors used ChatGPT (OpenAI) to assist with language editing and grammatical refinement. After using these tools, the authors carefully reviewed and revised the manuscript as needed and take full responsibility for the accuracy, integrity, and originality of the content of this publication.
